# Caries experience and oral health related quality of life in a cohort of Ugandan HIV-1 exposed uninfected children compared with a matched cohort of HIV unexposed uninfected children

**DOI:** 10.1186/s12889-020-08564-1

**Published:** 2020-03-30

**Authors:** Nancy Birungi, Lars T. Fadnes, Ingunn M. S. Engebretsen, Stein Atle Lie, James K. Tumwine, Anne Nordrehaug Åstrøm, for the ANRS 12174 and 12341 study groups

**Affiliations:** 1 Department of Clinical Dentistry, University of Bergen, P. O Box 7800 5020, Bergen, Norway; 2 Bergen Addiction Research Group, Department of Addiction Medicine, Haukeland University Hospital, Bergen, Norway; 3 Department of Global Health and Primary Health Care, University of Bergen, Bergen, Norway; 4 Department of Paediatrics and Child Health, School of Medicine, College of Health Sciences, Makerere University, Kampala, Uganda

**Keywords:** Dental caries, Oral health, Quality of life, HIV unexposed infected, Children

## Abstract

**Background:**

Very few studies consider the oral health status and quality of life in HIV-1 exposed uninfected (HEU) children. The aim of this study was to estimate the prevalence of caries in primary teeth and its oral health related quality of life impacts in HEU children compared to HIV-unexposed-uninfected (HUU) children, whilst adjusting for confounding covariates.

**Methods:**

This study uses data from the Ugandan site of the ANRS 121741 PROMISE- PEP trial (ClinicalTrials.gov, number NCT00640263) conducted in 2009–2013 that recruited mothers with HIV-1 and their uninfected children. Of 244 HEU-children-caretaker pairs available at the end of the one-year trial, 166 were re-enrolled in the ANRS 12341 PROMISE-PEP M&S study at 5–7 years and 164 were included in this study. These were age and sex-matched with 181 HUU children-caretaker comparators. Caries experience was recorded using World Health Organization’s Decayed, Missed and Filled teeth (dmft/DMFT) indices. The Early Childhood Oral health Impact Scale (ECOHIS) was used for assessment of oral health related quality of life. Mixed effects logistic regression was conducted with dmft and ECOHIS scores as outcomes and HIV-1 exposure status as the main exposure.

**Results:**

Forty-eight percent of HEU children and 60% of HUU had dmft> 0. Corresponding figures for ECOHIS> 0 were 12% of HEU and 22% of HUU. The crude analysis showed differences related to HIV-1 exposure in caries experience and oral health related quality of life. Mixed effect logistic regression analyses were not significant when adjusted for use of dental care and toothache. If caregivers’ DMFT> 0, the adjusted odds ratio for caries experience (dmft> 0) was 1.6 (95% CI: 1.0–2.8) while if dmft> 0 the adjusted odds ratio for quality of life impacts (ECOHIS> 0) was 4.6 (95% CI: 2.0–10.6).

**Conclusion:**

The prevalence of untreated caries in primary teeth and quality of life impacts was high in this study population. HIV-1 exposed uninfected children were not more likely than HUU children to experience dental caries or have impaired oral health related quality of life. Given the global expansion of the HEU child population, the present findings indicating no adverse effect of pre- and post-natal HIV-1 exposure on caries in deciduous teeth are reassuring.

## Background

According to the Global Burden of Disease 2010 study, untreated caries in deciduous teeth or early childhood caries (ECC) ranks the 10th most prevalent condition affecting 9% of the global child population [1]. In high income countries dental caries is among the most common chronic infectious diseases in children with prevalence reported up to 50%, whereas in low-income countries, caries in the primary dentition vary from 6 to 71% [2]. ECC leads to temporary pain and impacts on the quality of life of the child and family, including having financial and health implications [3].

Previous studies have confirmed that disadvantaged social status, exposure to adverse family environments and chronic diseases at early life stages constitute risk factors for developing dental caries in the primary dentition. Caretaker characteristics, such as poor feeding and rearing practices, parents’ own caries situation and poor oral hygiene associate with dental caries in the primary dentition of their offspring [4–8]. The increased risk of dental caries, oral mucosal lesions and periodontal disease in populations with HIV-1 has been thoroughly documented [9–11]. Despite experience with antiretroviral therapy globally, caries prevalence of children with HIV-1 vary with prevalence rates of 85 and 20% in low- and high income countries, respectively [12, 13]. During recent decades, industrialized countries have reported on a decline in children’s caries experience. In contrast, caries prevalence among children with HIV-1 have remained high in low- and high income countries [12, 14].

One important route of HIV-1 infection is transmission from mother to child accounting for 15–45% of transmissions without interventions. In sub Saharan Africa, implementation of antiretroviral therapy, ART, and improved counselling of postnatal risk factors such as infant feeding has dramatically reduced the number of infants being infected [15]. Even though the global plan to move towards elimination of new HIV-1 infections among children by 2015, was not achieved, tremendous success has been made in lowering the number of infants with HIV-1 [16]. Globally, between 2000 and 2014, the number of children acquiring HIV-1fell by 58% to 220,000 per year [16]. Concerted efforts coupled with increasing availability of effective prevention of mother-to-child treatment (PMTCT) interventions, make it possible to eliminate mother- to-child transmission in resource limited setting [17]. Since the coverage of effective interventions for mother to child transmission of HIV-1 is increasing faster than the antenatal HIV-1prevalence declines, a population of HIV exposed uninfected children (HEU) is emerging [18]. Every year above one million infants are born to women with HIV-1 worldwide, the majority of them in low income countries, and most of them will not become infected [19].

Studies from various settings have reported that HIV exposed, uninfected children (HEU) have poorer health outcomes than HIV unexposed uninfected children (HUU) [19–23]. Thus, HEU children seem to be disadvantaged with respect to birth outcomes such as preterm birth, linear growth, metabolism, adaptive and innate immune systems; and increased infectious morbidity [24]. In addition to direct exposure in utero and during delivery, mothers with HIV-1 can also affect the health situation of their children indirectly through poverty, caregiver insufficiency, unemployment, illness and bereavement [25]. Previous studies have shown that compared to HEU children, children with HIV infection have more negative impacts on their quality of life [26–28]. However, comparative studies for the quality of life of HEU children with HUU children are limited. Two studies from Uganda showed similar quality of life profiles among HUU and HEU children [27, 29]. Another study from Uganda found that infection/ exposure predicted low self-esteem and diminished positive outlook in the long term [28]. However, none of those studies investigated oral health related quality of life, specifically. Although HEU children are recognized to have specific health needs, very limited evidence is available regarding their survival, health, and oral health situation [14]. In addition, many cohort studies suffer from limited follow-up, lack of comparable control groups, inadequate consideration of confounding factors and inconsistent use of standardized and validated assessment tools [20].

This study compares caries in primary teeth and oral health related quality of life between a cohort of HEU-children at age 5–7 years who received peri-exposure prophylaxis during the initial breastfeeding period after birth and an age- and sex- matched control group of HIV unexposed uninfected children. Thus, the aim was to estimate the prevalence of caries in deciduous teeth and its oral health related quality of life impacts in HIV exposed uninfected children as compared to HIV unexposed uninfected children whilst adjusting for confounding covariates.

## Method

This study is based on data generated from the follow-up of the clinical HIV-1 peri-exposure prophylaxis with lopinavir-ritonavir ANRS12174 PROMISE- PEP trial (ClinicalTrials.gov, number NCT00640263) [30]. The ANRS12174 PROMISE- PEP trial is described in detail in a previous paper [30]. The trial conducted between 2009 and 2013, was a multi-center randomized trial including pregnant women with HIV-1, recruited at gestational age of 28–40 weeks at antenatal clinics in four African sites; Ouagadougou, Burkina Faso; East London, South Africa; Mbale, Eastern Uganda; and Lusaka, Zambia. HIV-1 infected pregnant women intending to breastfeed were referred for further assessment of inclusion criteria and again with their HIV uninfected children for enrolment and randomization at day 7 post-partum. Infants were eligible for inclusion if they were: a singleton; breastfed at day seven by their mothers; had a negative HIV-1 DNA PCR and had received any PMTCT. Further inclusion criteria was mother aged 18 years or older, intending to continue breastfeeding, HIV-1 infected, and not eligible for ART (either clinically or because CD4 count > 350 cells/μL at that time). All eligible mothers and infants followed the routine national mother-to-child transmission prophylaxis (PMTCT) with antepartum zidovudine, intrapartum nevirapine, zidovudine-lamivudine for mothers and nevirapine for infants for 7 days postpartum.

In Uganda, 278, seven-day old uninfected children born to HIV-1 infected women, were randomized to receive infant prophylaxis (either lamivudine, 3TC or boosted lopinavir-ritonavir, LPV/r daily) throughout the breastfeeding period from day 7 to 50 weeks. The primary outcome was mother to child HIV-1 transmission, diagnosed every 3 months with HIV-1 DNA PCR between 7 days and 50 weeks post-delivery. Infection rates, and clinical and biological severe adverse events did not differ between the two drug regimens suggesting that infant HIV-1 prophylaxis with either drug was not superior as both led to very low rates of HIV-1 postnatal transmission during 50 weeks of breastfeeding [30].

In 2017, 244 out of 278 mothers with HIV-1 infection and their uninfected children were eligible for re-enrollment in the follow-up study; the PROMISE-PEP Mechanism Safety study (PROMISE-PEP M&S Ritonavir ANRS12341). Of the 166 HEU children re-enrolled, 2 were excluded due to HIV-1 conversion. Thus, 68% of the eligible cohort of HEU children (164/244) was followed up with 32% (*n* = 112) missed due to attrition. A comparison group of 199 HUU children matched on age and sex, as well as their HIV uninfected mothers, were recruited from communities located in Mbale, Eastern Uganda; which was the site for the ANRS12174 PROMISE- PEP trial. Of the 199 HUU control children 19 were excluded due to a positive HIV-1 test result, leaving 181 HUU children and their uninfected mothers enrolled. This study uses information from interviews and clinical oral examinations of 164 HIV-1 infected caregiver-HEU child pairs and 181 age-and sex- matched HUU children and their caregivers at the follow-up in 2017.

### Interviews with mothers of the HEU and HUU children at follow-up

Trained interviewers performed face-to-face interviews with caregivers before the children underwent oral clinical examination using semi-structured interviews in the local language Lumasaba. The interview was constructed in English and translated into Lumasaba for use in the field. The schedule had been reviewed previously by project staff for semantic, experiental and conceptual equivalence of the source version. Sensitivity to culture and selection of appropriate words were considered [31]. Caregivers responded to questions about themselves and their children. Information was documented on case record forms (CRFs) and electronically with Capture software System.(Clinsight) and Epidata progaram www.epidata.dk for the clinical oral examinations.

Socio-demographic characteristics of caregivers were assessed in terms of level of education, type of income and marital status. Level of education was categorized into: did not finish primary school (1), end of primary school (2) higher education (3). Marital status was categorized: divorced (1), cohabiting/married (2), single (3), widowed (4) and recoded into single/divorced/widow (0) and married/cohabiting (1). Mother’s behavioral characteristics were assessed in terms of tooth brushing frequency (Categories of the socio-demographic and behavioral covariates are shown in Table 1). Type of income was categorized into (1) no regular income (2) regular income. Child’s characteristics were assessed in terms of dental care utilization, tooth brushing, ever toothache, and breastfeeding duration.

**Table 1:** Socio-demographic- clinical and behavioral characteristics of mother and child by child’s HIV-1 status at follow-up in the Ugandan part of the PROMISE PEP M&S study

	HIV- exposed uninfected (*n* = 164)	HIV- unexposed uninfected (*n* = 181)	Total (*n* = 345)
**Child characteristics**	% (*n*)	% (*n*)	% (*n*)
*Sex*			
Male	51 (84)	50 (89)	51 (175)
Female	49 (80)	50 (92)	49 (170)
*Age*			
5 year	28 (46)	31 (56)	29 (29)
6–7 year	72 (118)	69 (125)	70 (243)
*Ever taken to dental care*			
No	94 (155)	86 (156)	90 (31)
Yes	5 (9)^a^	14 (25)	10 (34)
*Toothbrush*			
Less once a day	20 (33)	10 (17)	15 (50)
Once a day or more	80 (133)^b^	90 (160)	85 (292)
*Ever toothache*			
No	90 (147)	79 (143)	84 (291)
Yes	11 (17)^b^	21 (38)	16 (55)
*Breastfeeding duration*			
0–6 months	31 (51)	3 (5)	16 (56)
7–12 months	65 (106)	13 (24)	38 (130)
> 12 months	4.(7)^b^	84 (149)	46 (156)
**Mother /caretaker characteristics**			
*Age*			
*18-32 yr*	40 (58)	58 (104)	50 (162)
*33+*	60 (90)^b^	42 (76)	50 (166)
*Toothbrush*			
No	59 (97)	44 (78)	52 (176)
Yes	40 (66)^b^	56 (99)	48 (165)
*Maternal CD4 count at birth*			
*> 500 cells/mm3*	98 (60)	–	
*≤ 500 cells mm3*	66 (40)	–	
*Marital status*			
Single/divorced, widow	26 (43)	18 (33)	22 (76)
Married/cohabiting	74 (121)^a^	82 (147)	78 (268)
*Type of income*			
No regular	60 (98)	72 (130)	66 (229)
Regular	40 (65)^a^	28 (51)	34 (116)
*Educational level*			
Primary school	38 (54)	40 (65)	39 (120)
End of primary school	20 (28)	22 (37)	21 (65)
Middle school/high school/college	42 (59)	38 (63)	40 (122)

^a^*p* < 0.05; χ^2^test, ^b^*p* < 0.001; χ^2^test

*Child impact section,* (CIS) of the early childhood oral health impact scale (ECOHIS) scale for child’s oral health related quality of life was assessed using six of its original nine questions [32]. The ECOHIS scale has previously been tested for its psychometric properties in the context of Ugandan pre-school children [33]. Caregivers were asked “has your child: ever had tooth ache, ever had swollen/bleeding gums, ever cried because of pain in mouth, ever failed to sleep because of pain in mouth, ever refused to eat because of pain in mouth, and ever refused to play because of pain in mouth. Dummy variables (0 = no, 1 = yes) were summed into a count variable (range 0–6) and dichotomized into 0 = no child impacts, 1 = at least one child impact. *Family impact section,* FIS, was assessed using the four original questions. “Because of child’s dental problems, how often have you or the other parent: taken time off from work, been upset, felt guilty and had financial problems? Response categories were rated on a 5 point scale from 0 = never to 4 = almost daily. Each item was dichotomized and the dummy variables were summarized into a count variable (range 0–4). The sum score was dichotomized into 0 = no family impacts and 1 = at least one family impact. A total ECOHIS score was constructed by adding the Child impact- and Family impact scores.

### Clinical oral examination of children and mothers at follow up

Two experienced and calibrated dental surgeons (NB and MM) performed a full-mouth oral clinical examination among children and their caregivers. Duplicate examinations, for inter-rater and intra-rater reliability were performed with 26 children not part of the ANRS12341-PROMISE- PEP trial cohort.

Dental caries was assessed on fully erupted primary/permanent teeth in children and caregivers using the decayed, missing, and filled teeth indexes (dmft/DMFT) in accordance with the World Health Organization (WHO) guidelines for field conditions [34]. A tooth was documented as decayed if it was visually cavitated with the aid of a dental mirror and periodontal probe (Michigan O probe), and recorded as missing when extracted due to caries, as confirmed by the caregiver. For analyses, dental caries was denoted in two ways: presence or absence of dmft/DMFT and the total number of decayed, missed and filled teeth in caregivers and their children with dmft/DMFT scored 0 and 1 for presence /absence of caries experience in children and caregivers. Number of erupted (fully emerged recorded as free occlusal /incisal surface) permanent teeth and number of primary teeth maintained were counted for children with mixed dentition. Since dental caries in the permanent dentition of children was very limited, DMFT was not calculated for children’s permanent teeth.

### Maternal and child HIV-1 status at baseline and follow-up

HIV-1 status of the HEU children was assessed using the HIV-1 DNA polymerase chain reaction from dried bloodspots. Mothers and children in the comparison group were tested for their HIV-1 status using serial and parallel HIV rapid testing with Determine, Stat-Pak and Uni-Gold, three test algorithm as recommended by the Ugandan Ministry of Health [35].

### Anthropometric status for caregiver-child pairs at follow -up

At the 5 year follow-up, anthropometric measurements for all the participants (caregivers - child pairs) in terms of weight and height were collected twice according to WHO guidelines (http://www.who.int/childgrowth/training/en/) using Seca- brand scales and Stadiometers to the nearest decimal place.

### Statistical analysis

STATA 15 (College Station, Texas 77,845 USA) was used for data analysis. Chi-square tests for (categorical) variables and independent sample T-tests (continuous variables) were used to assess differences in baseline characteristics between HEU children lost to follow-up and those who retained in the cohort, and for the crude associations between HIV-1 exposure status, covariates and the outcome variables. The data presented had a clustered two-level hierarchical structure with individuals clustered as matched HEU/HUU pairs. Ignoring that observations in a cluster are correlated (that is intra cluster dependency) usually leads to an underestimation of the standard errors, too narrow confidence intervals and higher Type 1 error rates. As clustering in these data were considered quite limited, multiple variable analyses were conducted with ordinary logistic regression, OLR, providing population averaged estimates. Secondly, multilevel random intercept logistic models (RIM) were fitted using mixed effects logistic regression. The RIM model explicitly allows for clustering by including both intra- and inter cluster variation in the model. Behavioral and clinical covariates statistically significantly associated both with HIV-1 exposure status and the outcomes of dental caries and oral quality of life were included in the multilevel logistic regression models as potential confounding variables. The effect of clustering (HEU/HUU pairs) was assessed by calculating intra class correlation coefficients (ICC) expressing variations between clusters as a proportion of the total variance (within plus between cluster variance). ICC varies from 0, which implies independent observations within cluster to 1 indicating no within cluster variation. Hence, high values of ICC implies dependency between observations within cluster. A likelihood –ratio test was calculated to test the null hypothesis that ICC equals 0. Rejection of the null hypothesis implies that the multilevel model is preferable.

## Results

Cohort participants (HEU children and their mothers with HIV) followed up (*n* = 164) did not differ significantly from those lost to attrition (*n* = 80) regarding socio-demographic- and behavioral characteristics at baseline, 7 days post-partum. More than 90% of the caregivers of HEU and HUU children included in the follow-up study were mothers. More than half (60%) of HIV-1 infected mothers followed up presented with CD4 counts > 500 cells /μL (median 524, IQR 439–515) at baseline. The calibration exercise comparing dmft scores for each primary tooth within and between examiners revealed intra- and inter-rater reliability of median Kappa (interquartile range-IQR) of 0.6 (0.5–0.7) and 0.7 (0.5–0.8), respectively. The corresponding scores for the DMFT of caregivers were 0.7 (0.5–0.9) and 0.6 (0.4–0.8), respectively.

Table 1 depicts the distribution of maternal- and child’s socio-demographic- and behavioral characteristics at follow-up according to child’s HIV-1 exposure status. A total of 50% of the HUU children were females and 69% were 5–7 years old (Table 1). Corresponding figures for HEU children were 49 and 72%, respectively. HEU children were more likely to have older caretakers (33 years and above) than their uninfected counterparts (60% versus 42% *p* < 0.0001), and more likely to have caretakers that were of single or divorced marital status (26% versus 18% *p* < 0.05). Regular income was more prevalent in the HEU group compared to the HUU group (40% versus 28% p < 0.05). Breastfeeding for more than 12 months was more common among HUU children than among their HEU counterparts (84% versus 4%, *p* < 0.001). Tooth brushing was most prevalent among HUU children and more prevalent among uninfected than among infected mothers.

As shown in Tables 2, 48% of HEU children had at least one missed, filled or decayed primary tooth (dmft> 0) and at least one tooth with untreated caries (dt > 0). The corresponding figure among HUU children was 60 and 57%, (*p* < 0.001). The prevalence of permanent decayed teeth (DT) was 4 and 5 in HEU and HUU children, respectively. The prevalence of caries experience (DMFT> 0) in HIV-1 infected and uninfected mothers was 81 and 71%, *p* < 0.001. Corresponding figures for mean DMFT were 4.6 and 2.8. The mean number of erupted permanent teeth in HEU and HUU children was respectively, 4.2 and 6.3 (*p* < 0.001). Corresponding figures regarding remained primary teeth were 17.4 and 14.7 (p < 0.001). Children’s anthropometric measures in terms of WAZ, HAZ and BAZ did not differ according to children’s HIV-1exposure status.

**Table 2:** Dental caries experience and anthropometric status of mother and child by child’s HIV-1 exposure status at follow-up in the Ugandan part of the PROMISE PEP M&S study

	HIV- exposed uninfected (*n* = 164) % (*n*)	HIV- unexposed uninfected (*n* = 181) % (*n*)
**Children**		
dmft^c^ > 0% (n)	48 (79)^a^	60 (109)
Mean dmft^c^ (sd)	2.0 (3.1)	2.1 (2.7)
dt > 0% (n)	48 (77)	57 (104)
Mean dt (sd)^d^	1.9 (2.8)	1.9 (2.5)
DT > 0% (n)	4 (7)	5 (10)
No of erupted permanent teeth mean (sd)	4.2 (3.8)^b^	6.3 (5.8)
No of maintained primary teeth mean (sd)	17.4 (7.2)^b^	14.7 (7.5)
WAZ^e^ weight for age (sd)^d^	−0.71 (1.1)	−0.70 (1.2)
HAZ^f^ height for age (sd)^d^	−0.72 (2.0)	−0.51 (1.8)
WHZ^g^ weight for height (sd)^d^	−0.30 (1.7)	−0.53 (1.1)
**Mothers**		
DMFT^c^ > 0% (n)	81 (133)^b^	71 (129)
Mean DMFT^c^ (sd)^d^	4.6 (5.3)^b^	2.8 (3.2)

^a^*p* < 0.05;χ^2^test,^b^*p* < 0.001 χ^2^test,^c^ dmft/DMFT- Decayed, missing, filled teeth, ^d^ standard deviation ^e^ weight for age z score, ^f^ height for age z score, ^g^ weight for height z score

Table 3 depicts responses to the ECOHIS inventory according to each separate item in the total study group as well as among HEU and HUU children. As shown, the prevalence of child impacts (CIS) amounted to 12 and 22% (p < 0.001) in HEU and HUU children, respectively. Corresponding figures for FIS and total ECOHIS scores were 6% versus 8 and 12% versus 22%. The prevalence of each separate CIS and FIS impact was consistently higher among HUU children than among their HEU counterparts. The internal consistency reliability in terms of Cronbach’s alpha was 0.95 (CIS score), 0.86 (FIS score) and 0.93 (Total ECOHIS score).

**Table 3:** Frequency distribution and Cronbach’s alpha of child impact-, family impact-, and total scores by HIV-1 status at follow –up in the Ugandan part of PROMISE PEP M&S study

Items	Total % (*n*)	HIV-1exposed uninfected % (*n*)	HIV- unexposed uninfected % (*n*)
**Child impact- Has child:**			
Ever had toothache	16 (55)	10 (17)^a^	21 (38)
Ever had swollen/bleeding gums	10 (35)	7 (11)^a^	13 (24)
Ever cried because of pain in mouth	12 (41)	9 (15)	14 (26)
Ever failed to sleep because of pain in mouth	10 (33)	7 (12)	11 (21)
*Ever refused to eat because of pain in mouth*	10 (36)	8 (13)	13 (23)
Ever refused to play	8 (29)	5 (8)^a^	1 (21)
***Family impact***			
How often have you or another family member because of problems with child’s mouth and teeth-----			
Taken time off work	6 (20)	7 (11)	5. (9)
Been upset	4 (13)	2 (4)	5 (9)
Felt guilty	5 (16)	3 (5)	6 (11)
Had financial difficulties	3 (9)	2 (4)	3 (5)
	Cronbach’s alpha		
**Child impact score (CIS) > 0**	0.95	12 (19)^a^	22 (40)
**Family impact score (FIS) > 0**	0.86	6 (10)	8 (14)
**ECOHIS total score > 0**	0.93	12 (20)^a^	22 (38)

^a^*p* < 0.05;χ^2^test

Table 4 depicts odds ratios (OR) estimates and 95% confidence intervals for dental caries experience (dmft> 0) in primary teeth by HIV-1 exposure status and covariates in unadjusted and adjusted analyses. No crude association occurred between caries experience and socio-demographic factors except for type of income. Caries experience differed statistically significantly between exposed and unexposed children with 48 and 60% having dmft> 0, respectively. Child’s toothache, child’s experience with dental care and caretakers’ caries experience associated statistically significantly with children’s caries experience as well as their HIV-1 exposure status and were included as possible confounding factors in the multiple variable logistic regression models. Mixed model logistic regression revealed that HEU children were not more likely than HUU children to have dental caries (OR 0.7, 95% CI: 0.4–1.2). Cluster specific ORs based on mixed model logistic regression revealed the following significant covariates; ever taken to dental care (OR 3.1, 95% CI: 1.1–8.9), ever experienced toothache (OR 7.8, 95% CI: 2.9–20.7), mothers’ tooth brushing (OR 1.6, 95% CI: 1.0–2.6), mothers’ DMFT (OR 1.6, 95% CI: 1.0–2.8) and regular income (OR 1.7, 95% CI: 1.0–2.8). No two-way interaction occurred between HIV-1 exposure status and other covariates (Supplementary Table 1, Additional file 1).

**Table 4:** Children’s dental caries experience (dmft> 0) by HIV.1 exposure status socio-demographic- clinical and behavioral characteristics. Unadjusted and adjusted ordinary logistic regression and mixed model logistic regression

Child characteristics	Cross tabulation	OLS^c^ dmft> 0	Adjusted OLS^c^ dmft> 0	Adjusted RIM^d^ dmft> 0
**HIV-1Status**	% (n)	OR (95% CI)^e^	OR (95% CI)^e^	OR (95% CI)^e^
*HIV-1 unexposeed uninfected*	60 (109)	1	1	1
*HIV exposed uninfected*	48 (79)^a^	0.6 (0.4–0.9)^f^	0.8 (0.4–1.2)	0.7 (0.4–1.2)
*Sex*				
Male	58 (102)	1		
Female	51 (87)	0.8 (0.5–1.2)		
*Age*				
5 year	55 (56)	1		
6-7 year	54 (132)	1.0 (0.6–1.5)		
*Ever taken to dental care*				
No	51 (159)	1	1	1
Yes	85 (29)^b^	5.5 (2.0–14.7)^g^	3.1 (1.1–8.9)	3.1 (1.1–8.9)
*Ever toothache*				
No	48 (139)	1	1	1
Yes	89 (49)^b^	8.9 (3.7–21.3)^g^	7.8 (2.9–20.6)	7.8 (3.0–20.7)
*Breastfeeding duration*				
0–6 months	54 (30)	1		
7–12 months	50 (65)	0.9 (0.5–1.6)		
> 12 months	59 (92)	1.2 (0.7–2.3)		
**Mother /caretaker characteristics**				
*Tooth brushing*				
No	49 (86)	1	1	1
Yes	60 (99)^a^	1.5 (1.0–2.4)^f^	1.6 (1.0–2.8)	1.6 (1.0–2.6)
*Caries experience*				
*DMFT = 0*	44 (37)	1	1	1
*DMFT > 0*	58 (151)^a^	1.7 (1.0–2.8)^f^	1.6 (1.0–2.8)	1.6 (1.0–2.8)
*Marital status*				
Single/divorced, widow	55 (42)	1		
Married/cohabiting	54 (145)	1.0 (0.6–1.6)		
*Type of income*				
No regular	51 (116)	1	1	1
Regular	62 (72)^a^	1.6 (1.0–2.5)^f^	1.7 (1.0–2.8)	1.7 (1.0–2.8)
*Educational level*				
No- end of Primary school	60 (184)	1		
Middle/high school	40 (122)	1.1 (0.7–1.7)		

Intra class correlation ~ 0

*Dmft/DMFT* Decayed, missing, filled teeth ^a^*p* < 0.05;χ^2^test,^b^*p* < 0.001;χ^2^test, ^c^ Ordinary least square, ^d^ Random intercept model, ^e^ Odds ratio (95% confidence interval), ^f^*p* < 0.05,^g^*p* < 0.001

Table 5 depicts the unadjusted and adjusted associations from cross tabulation, ordinary logistic regression analyses and mixed model logistic regression regarding the total ECOHIS scores by HIV-1 status and potentially confounding covariates. ECOHIS scores differed statistically significantly between exposed and unexposed children with respectively, 12 and 22% having ECOHIS score > 0. Adjusted cluster specific ORs based on mixed model analyses was 4.6 (95% CI: 2.0–10.6) regarding child’s caries experience and 5.0 (95% CI 1.8–13.6) regarding ever been taken to dental care. HIV-1 exposure status and breastfeeding duration did not remain statistically significantly associated with ECOHIS scores in adjusted multiple variable regression models. There were no significant interactions observed (See supplementary Table 2, Additional file 1). The intra class correlation (95% CI) estimated was limited amounting to ICC (95% CI: 0.2, (0.02–0.73) implying that 20% of the variance in ECOHIS scores explained by covariates was between clusters and 80% within clusters.

**Table 5:** Child- and family oral health related quality of life scores (ECOHIS> 0) by HIV-1 exposure status, socio-demographic –clinical and behavioral factors. Unadjusted and adjusted ordinary logistic regression and mixed model logistic regression

Child characteristics	Unadjusted	Univariate OLR^c^	Adjusted OLR^c^	Adjusted RIM^d^
**HIV-status**	% (n)	OR (95% CI)^e^	OR (95% CI)^e^	OR (95% CI)^e^
*HIV unexposed- uninfected*	22 (38)	1	1	1
*HIV exposed uninfected*	12 (20)^a^	0.5 (0.3–0.9)^a^	1.5 (0.5–4.4)	1.4 (0.4–4.8)
*Sex*				
Male	17 (29)	1		
Female	18 (29)	1.1 (0.6–1.9)		
*Age*				
5 year	13 (13)	1		
6–7 year	19 (46)	1.6 (0.8–3.1)		
*Ever taken to dental care*				
No	14 (42)	1	1	1
Yes	48 (16)^b^	5.9 (2.8–12.6)^f^	4.2 (1.9–9.3)	5.0 (1.8–13.6)
*Caries experience*				
dmft = 0	7 (10)	1	1	1
dmft> 0	26 (48)^b^	5.2 (2.5–10.7)^f^	4.3 (2.0–9.1)	4.6 (2.0–10.6)
*Breastfeeding duration*				
0–6 months	14 (8)	1	1	1
7–12 months	11 (14)	0.7 (0.3–1.9)	0.7 (0.3–2.0)	0.8 (0.3–2.3)
> 12 months	23 (36)^a^	1.8 (0.8–4.3)^g^	2.2 (0.6–8.3)	2.3 (0.5–10.0)
**Mother /caretaker characteristics**				
*Tooth brushing*				
No	17 (28)	1		
Yes	18 (29)	1.1 (0.6–1.9)		
*Caries experience*				
*DMFT = 0*	13 (11)	1		
*DMFT > 0*	19 (48)	1.6 (0.8–3.4)		
*Marital status*				
Single/divorced, widow	13 (10)	1		
Married/cohabiting	19 (48)	1.5 (0.7–3.0)		
*Type of income*				
No regular	15 (33)	1		
Regular	21 (24)	1.6 (0.9–2.8)		
*Educational level*				
Primary school	19 (23)	1		
End of primary school	15 (10)	0.8 (0.4–1.8)		
Middle/high school	19 (23)	1.0 (0.5–1.8)		

Intra class correlation (95% CI); 0.2 (0.0–0.7)

*dmft/DMFT* Decayed, missing, filled teeth ^a^*p* < 0.05; χ ^2^test, ^b^ p < 0.001;χ^2^test, ^c^ Ordinary least square, ^d^ Random intercept model, ^e^ Odds ratio (95% confidence interval), ^f^*p* < 0.001, ^g^*p* < 0.05

## Discussion

This study is among the first to report on caries in primary teeth and its oral health related quality of life impacts of 5–7 year-old HEU children as compared to their HUU counterparts living in a similar non-occidental cultural setting. At the time of the present study, no other published study comparing both clinical- and subjective oral health indicators of HEU and HUU children was available for comparison. The prevalence of untreated caries and quality of life impacts was high in this study population with 60 and 48% of respectively, HUU and HEU children showing untreated caries and 22% HUU and 12% HEU children reporting oral health related quality of life impacts. Compared with HUU community controls, the present study showed non-significant odds of caries experience and ECOHIS scores in HEU children who were breastfed under protection of peri-exposure prophylaxis for a postnatal period of 50 weeks. Nevertheless, independent of children’s HIV-1 exposure status, the odds of children’s caries experience increased with caretakers’ own caries experience and regular income status. Similarly, odds ratios of having quality of life impacts increased with child’s caries experience and frequency of dental care visiting.

Strengths of this study include its nested long-term cohort design regarding the HEU children, and its use of a matched group of community HUU comparators. Previous studies have been criticized of using control groups of HUU children that differ significantly in socioeconomic status or breastfeeding patterns, thus leading to serious confounding [19, 20]. Contrary to the situation in many high income countries where most HEU children come from marginalized sectors of the society, HIV-1 epidemic in low income countries is more generalized across the socioeconomic spectrum and thus HEU and HUU children are socio-economically more comparable [19]. Another strength is the strict characterization of the HIV-1 status of mothers and their children as well as use of a validated measure of ECOHIS scores. Statistical models for cluster adjustment are not well established in dental public health research [36, 37]. Thus, the use of novel statistical methods in the present study stands as a methodological contribution to the scientific literature by accounting for the clustered structure in matched group data. The main limitation is the difference in follow-up between HEU and HUU children through lack of a concomitant control group. Missing data and loss to follow-up might also limit the interpretation of the findings as the present analyses might suffer from being underpowered. It is important to note that the caries prevalence in primary teeth of the HEU children might have been underestimated as only children passively followed up were included in the analyses and those who might have died before follow-up examination were not part of the group analyzed. Low statistical power due to the limited sample size available at follow-up may have been responsible for the absence of statistically significant association between HIV-1 exposure groups and dental caries as well as oral health related quality of life. Post-hoc power calculation was 61%. There also could have been.

The present findings did not support the expectation that pre- and post-natal HIV-1exposure adversely affect dental caries in primary teeth nor oral health related quality of life of 5–7 years old children in Uganda. In this aspect, the present study accords with some previous African studies but contrasts with others investigating aspects of HEU children’s general health and developmental delays [38–41]. The caries prevalence of 5–7-year-old HEU and HUU children observed in this study is comparable to that of children in the Ugandan general child population of similar age, previously estimated to range from 38 to 65% [31, 42]. Previous studies have reported on worse oral health among children with HIV-1 compared to healthy children of the same age group [11, 43]. Studies from low-income countries have reported on prevalence of caries in the deciduous teeth of children with HIV-1 ranging between 55 and 64%, which is comparable to the prevalence reported in some studies of uninfected children in Uganda as well as among the community controls participating in the present study [9, 44]. Recently, a study of children with HIV-1 and HIV-exposed uninfected children /adolescents in the United States showed that participants with HIV-1 were more likely to present with caries in primary teeth, whereas caries experience in the permanent dentition and periodontal disease markers did not differ between the two groups [14]. Another study from Nigeria involving children with HIV, HUU and HEU showed that HEU children might not have a different caries experience than HUU children [41]. In accordance with the findings of this study, a Ugandan study revealed that quality of life impacts were similar for perinatally HEU and HUU community controls [27].

The prevalence of dental caries and oral impact scores were higher among HUU children than among their HEU counterparts. On the other side there were indications of a minor developmental delay in eruption of permanent teeth among HEU children presenting with significantly higher number of non-exfoliated primary teeth than HUU children [45]. The present findings both accord with and contradict previous studies from sub-Saharan Africa reporting on increased risk of developmental delays and poor nutritional status among HEU as compared to HUU children [20–22]. Notably, studies indicate that before the universal availability of antiretroviral therapy, ART, HEU children had higher mortality-, morbidity and growth failure risk than HUU children, whereas later studies from the ART era show heterogeneous findings [22]. In a meta-analytic review of neurodevelopment in young children born to mothers with HIV, Mc Henry et al. [40] found that children with HIV-1 and HEU had worse health outcomes than their HUU counterparts and moreover, that HEU children without exposure to ART had worse health outcomes than their ART exposed counterparts. The HEU children in the present study were considered to be at low risk as they received infant prophylaxis (either lamivudine 3TC or boosted lopinavir/ritonavir, LPV/r daily) throughout the 50 weeks of breastfeeding in addition to standard prevention during delivery, complicating the natural history of HIV-1 exposure. In addition, the mothers with HIV-1 included were immune competent with CD 4 counts > 350 cells /μL when recruited into the cohort. Evidently, HEU children born to mothers with more severe disease assessed by the CD4 count present with higher rates of morbidity than those born to mothers with less sever disease [19, 20]. Moreover, ART related improvements in the immune function of the HEU children participating in this study might have reduced their infection susceptibility generally and thus their vulnerability to caries in the deciduous dentition [19, 20]. However, there might be other less measured extrinsic factors such as contact with the health system and years of counselling which may have altered help-seeking behavior and awareness around one’s own and the child’s health which may have influenced the behavior and ultimately the oral health. We have not controlled for all potential confounding factors.

Independent of children’s HIV-1 exposure, and consistent with previous studies from sub Saharan Africa, caries in primary teeth and oral health related quality of life varied significantly according to child- and caretakers’ clinical and behavioral characteristics [4, 8, 31, 33]. Thus, caretakers’ caries experience was strongly associated with the caries experience of their offspring [8, 33]. Several plausible mechanisms are available for the relationship between caretakers and children’s caries experience, such as mother to child transmission of genetic factors and shared oral habits between members of the whole family [8]. Finally, caries experience in HEU children was a strong covariate of their oral quality of life [40, 42]. This finding indicates that ECOHIS has satisfactory criterion validity when utilized in the context of HEU and HUU Ugandan children [8, 33].

## Conclusion

The prevalence of untreated caries in deciduous teeth and quality of life impacts was high in this study population. HIV-1 exposed uninfected children were not more likely than HUU children to experience dental caries or impaired oral health related quality of life. Given the global expansion of the HEU child population, the present findings indicating no adverse effect of pre- and post-natal HIV-1 exposure on caries in primary teeth are reassuring.

## Supplementary information

**Additional file 1.** Evaluation of effects of interaction of covariates with HIV-1 (EBF) exposure regressed with dental caries experience. Data indicating the individual association of the main exposure with dental caries experience as well as the effects of the main exposure and other covariates with dental caries experience.

## Data Availability

The datasets used and/or analysed during the current study are available from the corresponding author on reasonable request.

## Abbreviations

ARV: Antiretroviral
ART: Antiretroviral therapy
CI: Confidence interval
CIS: Child impact scale
ECC: Early childhood caries
ECOHIS: Early childhood oral health impact scale
DMFT/ dmft: Decayed missing filled teeth surfaces
FIS: Family impact scale
HAART: Highly active antiretroviral therapy
HIV: Human deficiency virus
HEU: HIV exposed uninfected
HUU: HIV unexposed uninfected
ICC: Inter class correlation
IQR: Interquartile range
OR: Odds ratio
OLR: Ordinary logistic regression
PMTCT: Prevention of mother to child transmission
PROMISE-PEP: Promoting infant health and nutrition in Sub-Saharan Africa: Safety and efficacy of infant Peri-Exposure Prophylaxis
PROMISE-PEP-M&S: Promoting infant health and nutrition in Sub-Saharan Africa: Safety and efficacy of infant Peri-Exposure Prophylaxis to prevent HIV-1 transmission by breastfeeding-Mechanisms & Safety
RIM: Random intercept model
WHO: World Health Organisation
