# Supplementary material for: Caries experience and oral health related quality of life in a cohort of Ugandan HIV-1 exposed uninfected children compared with a matched cohort of HIV unexposed uninfected children
Source: BMC Public Health. 2020 Mar 30;20:423. doi: 10.1186/s12889-020-08564-1 (PMC7106612; doi:10.1186/s12889-020-08564-1)
Supplement: Supplementary file 1 — Additional file 1. Evaluation of effects of interaction of covariates with HIV-1 (EBF) exposure regressed with dental caries experience. Data indicating the individual association of the main exposure with dental caries experience as well as the effects of the main exposure and other covariates with dental caries experience. [file 12889_2020_8564_MOESM1_ESM.docx]

**Table 1: Evaluation of effects of interaction of covariates with HIV exposure regressed with dental caries**

Marital status

| Variable | Incidence rate ratio | 95% confidence interval |
| --- | --- | --- |
| HIV exposure | 0.5 | 0.2−1.4 |
| Marital status | 0.8 | 0.4−1.8 |
| Marital status *HIV exposure | 1.2 | 0.4−3.4 |

Sex

| Variable | Incidence rate ratio | 95% confidence interval |
| --- | --- | --- |
| HIV exposure | 0.4 | 0.1–1.6 |
| Sex | 0.6 | 0.4–1.2 |
| Sex*HIV exposure | 1.3 | 0.5–3.0 |

Dental care

| Variable | Incidence rate ratio | 95% confidence interval |
| --- | --- | --- |
| HIV exposure | 0.7 | 0.4–1.1 |
| Dental care | 5.9 | 1.7–20.5 |
| Dental care*HIV exposure | 0.7 | 0.1–5.2 |

Months breastfed

| Variable | Incidence rate ratio | 95% confidence interval |
| --- | --- | --- |
| HIV exposure | 1.5(0.2−0.8) | 0.2−0.8 |
| Breastfeeding duration categories | 1.1 | 0.5–2.0 |
| Breastfeeding duration categories*HIV exposure | 0.6 | 0.2–1.5 |

Mother’s caries experience

| Variable | Incidence rate ratio | 95% confidence interval |
| --- | --- | --- |
| HIV exposure | 0.5 | 0.2–1.1 |
| Mothers caries experience | 1.7 | 0.9–3.2 |
| Mothers caries experience *HIV exposure | 1.3 | 0.5–3.8 |

Type of income

| Variable | Incidence rate ratio | 95% confidence interval |
| --- | --- | --- |
| HIV exposure | 1.4 | 0.4–5.1 |
| Type of income | 2.5 | 1.2–5.1 |
| Type of income *HIV exposure | 0.5 | 0.2–1.4 |

Education level

| Variable | Incidence rate ratio | 95% confidence interval |
| --- | --- | --- |
| HIV exposure | 0.7 | 0.2–2.7 |
| Education level | 1.1 | 0.6–2.1 |
| Education level*HIV exposure | 1.0 | 0.4–2.5 |

Age

| Variable | Incidence rate ratio | 95% confidence interval |
| --- | --- | --- |
| HIV exposure | 1.8 | 0.3–9.2 |
| Age | 1.3 | 0.7–2.5 |
| Age *HIV exposure | 0.5 | 0.2–1.4 |

Adult brush

| Variable | Incidence rate ratio | 95% confidence interval |
| --- | --- | --- |
| HIV exposure | 0.5 | 0.2–0.8 |
| Adult brushing | 1.0 | 0.6–1.9 |
| Adult brushing *HIV exposure | 2.1 | 0.9–4.9 |

ECOHIS

| Variable | Incidence rate ratio | 95% confidence interval |
| --- | --- | --- |
| HIV exposure | 0.7 | 0.4–1.1 |
| ECOHIS | 4.0 | 1.7–9.7 |
| ECOHIS*HIV exposure | 1.3 | 0.3–5.6 |

**Table 2: Evaluation of effects of interaction of covariates* with HIV (EBF) exposure regressed with oral health impacts**

Marital status

| Variable | Incidence rate ratio | 95% confidence interval |
| --- | --- | --- |
| HIV exposure | 2.0 | 0.32–12.31 |
| Marital status | 0.65 | 0.28–1.52 |
| Marital status *HIV exposure | 0.33 | 0.070–1.59 |

sex

| Variable | Incidence rate ratio | 95% confidence interval |
| --- | --- | --- |
| HIV exposure | 2.0 | 0.32–12.31 |
| Sex | 0.65 | 0.28–1.52 |
| Sex*HIV exposure | 0.33 | 0.070–1.59 |

| Variable | Incidence rate ratio | 95% confidence interval |
| --- | --- | --- |
| HIV exposure | 2.0 | 0.32–12.31 |
| Caregiver tooth brushing | 0.65 | 0.28–1.52 |
| Care giver tooth brushing *HIV exposure | 0.33 | 0.070–1.59 |

Breastfeeding duration

| Variable | Incidence rate ratio | 95% confidence interval |
| --- | --- | --- |
| HIV exposure | 2.0 | 0.32–12.31 |
| Breastfeeding duration categories | 0.65 | 0.28–1.52 |
| Breastfeeding duration categories*HIV exposure | 0.33 | 0.070–1.59 |

Mother’s caries experience

| Variable | Incidence rate ratio | 95% confidence interval |
| --- | --- | --- |
| HIV exposure | 2.0 | 0.32–12.31 |
| Mothers caries experience | 0.65 | 0.28–1.52 |
| Mothers caries experience *HIV exposure | 0.33 | 0.070–1.59 |

Type of income

| Variable | Incidence rate ratio | 95% confidence interval |
| --- | --- | --- |
| HIV exposure | 2.0 | 0.32–12.31 |
| Type of income | 0.65 | 0.28–1.52 |
| Type of income *HIV exposure | 0.33 | 0.070–1.59 |

Education level

| Variable | Incidence rate ratio | 95% confidence interval |
| --- | --- | --- |
| HIV exposure | 2.0 | 0.32–12.31 |
| Education level | 0.65 | 0.28–1.52 |
| Education level*HIV exposure | 0.33 | 0.070–1.59 |

Age

| Variable | Incidence rate ratio | 95% confidence interval |
| --- | --- | --- |
| HIV exposure | 2.0 | 0.32–12.31 |
| Age | 0.65 | 0.28–1.52 |
| Age *HIV exposure | 0.33 | 0.070–1.59 |

Dental care

| Variable | Incidence rate ratio | 95% confidence interval |
| --- | --- | --- |
| HIV exposure | 2.0 | 0.32–12.31 |
| Dental care | 0.65 | 0.28–1.52 |
| *Dental care*HIV exposure | 0.33 | 0.070–1.59 |
